# *Candidatus* Allocryptoplasma Godzilla, a Novel *Ca.* Allocryptoplasma Species Detected in Marine Iguanas from Galápagos Islands

**DOI:** 10.3390/pathogens15090892

**Published:** 2026-08-25

**Authors:** Ricardo G. Maggi, Emma Williams, Gregory A. Lewbart, Maximilian Hirschfeld, Kenneth J. Lohmann, Juan Pablo Muñoz-Pérez, Brittany S. Thomas, Bridget Appiah, Edward B. Breitschwerdt

**Affiliations:** 1 Department of Clinical Sciences, College of Veterinary Medicine, North Carolina State University, Raleigh, NC 27607, USA; egwill24@ncsu.edu (E.W.); galewbar@ncsu.edu (G.A.L.); bmsherbe@ncsu.edu (B.S.T.); bkappiah@ncsu.edu (B.A.); ebbreits@ncsu.edu (E.B.B.); 2 Galápagos Science Center (GSC), Universidad San Francisco de Quito (USFQ) and University of North Carolina at Chapel Hill, Puerto Baquerizo Moreno, Galápagos 200101, Ecuador; maxh@enphocus.net (M.H.); klohmann@email.unc.edu (K.J.L.); jmunozp@usfq.edu.ec (J.P.M.-P.); 3 Department of Biology, University of North Carolina at Chapel Hill, Chapel Hill, NC 27599, USA; 4 School of Science, Technology and Engineering, University of the Sunshine Coast UniSC, Fraser Coast161 Old Maryborough Road, Hervey Bay, QLD 4655, Australia

**Keywords:** *Candidatus* Allocryptoplasma godzilla, marine iguanas, lava lizards, ticks, vector-borne diseases, 16S ribosomal RNA, *sucA*, *rpoB*, Allocryptoplasma, anaplasmaceae

## Abstract

*Candidatus* (*Ca.*) Allocryptoplasma is a newly identified, genetically diverse genus of vector-borne bacteria closely related to established blood-borne zoonotic pathogens, including *Anaplasma*, *Ehrlichia*, and *Neoehrlichia* species. *Ca.* Allocryptoplasma is a globally distributed pathogen detected across taxonomically diverse tick species and varied ecological niches. It maintains a complex enzootic circulation involving multiple vertebrate classes, including birds, small mammals, primates, and particularly reptiles, which serve as reservoir hosts. Methods: To characterize this emerging agent, multi-locus genetic DNA sequence analyses using the maximum likelihood method were performed on 16S rRNA, *sucA*, and *rpoB* genes amplified from the blood and vectors of marine iguanas and lizard species sampled across multiple islands in the Galápagos archipelago. Results: The compiled genomic data demonstrate that the *Ca.* Allocryptoplasma detected in the Galápagos lizards and marine iguanas represents a distinct lineage and a novel *Ca.* Allocryptoplasma species. Conclusions: Given its broad host plasticity, widespread geographic footprint, and vector-mediated transmission, the potential of *Ca.* Allocryptoplasma as a hidden, emerging zoonotic blood pathogen warrants intensive further evaluation.

## 1. Introduction

The Anaplasmataceae comprises a family of small, Gram-negative, obligate intracellular bacteria belonging to the order Rickettsiales that infect a wide range of hosts worldwide, causing medically important zoonotic diseases in animals and human patients [1]. Historically, this family consisted of six key genera, including *Anaplasma*, *Ehrlichia*, *Neorickettsia*, *Wolbachia*, *Aegyptianella*, and *Candidatus* Neoehrlichia.

In recent years, a distinctive group of Anaplasmaceae species, designated *Candidatus* Allocryptoplasma, has been detected in ticks, mammals, birds, and reptiles worldwide [2]. As a putative vector-borne pathogen, *Ca.* Allocryptoplasma (initially described as *Ca.* Cryptoplasma) was first reported in *Ixodes pacificus* ticks from California in 2015 and subsequently in several tick species worldwide, including *I. ricinus* from France, Italy, Tunisia, Slovakia, and Morocco; *Amblyomma tholloni* and *H. parmata* from Uganda; *A. coelebs* from French Guiana; and *H. longicornis* from South Korea [2,3,4]. Molecular evidence, mostly based on DNA sequencing of the 16S rRNA gene, indicates that a wide range of vertebrates, including mammals (striped field mice, *Apodemus agrarius*), birds, and reptiles (including several lizard species), may act as primary hosts for the maintenance and enzootic circulation of *Ca.* Allocryptoplasma in nature [2,3,4,5]. Although *Ca.* Allocryptoplasma DNA has been amplified from the blood of birds, mammals and reptiles, cell tropism and pathogenic potential are as yet poorly understood. Because the organism has been found in numerous tick species and in potential reservoir hosts that span numerous vertebrate species, *Ca.* Allocryptoplasma are of increasing medical interest.

Marine iguanas (*Amblyrhynchus cristatus*) are considered one of the most remarkable examples of adaptive radiation and evolutionary specialization. As the only seagoing lizards, their unique biology offers critical insights into how organisms adapt to extreme environments through rapid genetic evolution, physiological plasticity, and environmental adaptation [6,7,8,9,10]. As endemic herbivores vital to the Galápagos ecosystem, marine iguana populations face significant threats from environmental hazards, including oil spills, plastic pollution, and pathogens introduced by invasive species.

In 2014, during a health assessment of wildlife across the Galápagos archipelago, our research group detected a previously unknown “Anaplasma-like” infection in blood and ticks from marine iguanas (*Amblyrhynchus cristatus*) on San Cristóbal Island (unpublished data). Initial 16S rRNA sequence analysis [11] showed a 95% homology (324/341 bp) to *Anaplasma phagocytophilum* (GenBank CP006618), though concurrent attempts to amplify the full gene or additional genetic markers were unsuccessful.

Later, a 2025 transcriptomic study by Uesseler et al. [4] used next-generation sequencing to identify *Ca.* Allocryptoplasma at a high prevalence in marine iguana blood samples across nine Galápagos islands. Since 16S rRNA sequence analysis is inadequate for resolving phylogenetic relationships at lower taxonomic levels [4], the present work describes a multilocus genotyping analysis using rpoB and sucA housekeeping genes to resolve species-level phylogenetic relationships in this group and to provide phylogenetic evidence supporting the formal recognition of this bacterium as a novel candidate species. To achieve this, biobanked marine iguanas’ blood samples from the 2014 study were analyzed using custom primers targeting three distinct genetic loci: the 16S ribosomal RNA (16S rRNA), alpha-ketoglutarate dehydrogenase (*sucA*), and RNA polymerase subunit beta (*rpoB*) genes [2,4].

## 2. Materials and Methods

Blood DNA from a total of 85 marine iguanas, collected from Isabela (*n* = 18), San Cristobal (*n* = 10), Santa Cruz (*n* = 10), Lobos (*n* = 33) and Española (*n* = 14), as well as DNA from ticks collected from lava lizards and marine iguanas from San Cristobal (from 29 and 4 individuals, respectively) were manually extracted using a DNeasy Blood and Tissue Kit (Qiagen, 19,300 Germantown Rd, Germantown, MD 20874, USA). DNA quantity and quality were assessed by spectrophotometry using a Fisher Nanodrop One Spectrophotometer (Fisher Scientific, Waltham, MA 02451, USA).

Primers for partial DNA amplification of the16S rRNA, *sucA*, and *rpoB* genes were developed in-house using sequences form target gene previously deposited in the GenBank database (16SrRNA Genbank accession numbers: MG924904, KP276585, KP276587, OQ724854-62, and OQ724839; *sucA*: GenBank accession numbers OQ724538-41, OQ724542 to OQ724551, and OQ724552; *rpoB*: GenBank accession numbers OQ724582 and KP276604) and analyzed using Clustal W multi-sequence alignment (AlignX, Vector NTI Advanced 10.3.0 from Invitrogen).

DNA amplification of the16S rRNA, *sucA*, and *rpoB* genes was performed in a 25 μL PCR reaction using 12.5 μL of SsoAdvanced Universal SYBR green Supermix (Bio-Rad, Hercules, CA 94547, USA), 0.2 μL of 100 μM of each forward and reverse primers described in Table 1 (IDT DNA Technology, Coralville, IA52241, USA), 7.5 μL of Ultra-Pure molecular grade water (Genesee Scientific, San Diego, CA, USA), and 5 μL of DNA template. PCR was performed in a CFXOpus (Bio-Rad, Hercules, CA 94547, USA) under the following conditions: a single hot-start cycle at 95 °C for 3 min followed by 45 cycles of denaturing at 94 °C for 10 s, annealing at 66 °C for 10 s, and extension at 72 °C for 30 s. Positive amplicons were analyzed by analysis of detectable fluorescence vs. cycle threshold values, as well as by melting curve (65 °C to 95 °C at 0.5 °C/s). Amplified products were purified and sequenced by Sanger’s method (GENEWIZ Inc., Raleigh, NC 27560, USA) and analyzed using Clustal W multi-sequence alignment (AlignX, Vector NTI Advanced 10.3.0 from Invitrogen) and compared to sequences previously deposited in the GenBank database [12].

Tick species identification (collected from lava lizards and marine iguanas) was performed by partial DNA amplification of the mitochondrial 12SrRNA small ribosomal DNA using methods previously reported with minor modifications [13]. Briefly, PCR was performed in a 25 μL reaction using 12.5 μL of SsoAdvanced Universal SYBR green Supermix (Bio-Rad, Hercules, CA, USA), 0.2 μL of 100 μM of each forward and reverse primers described in Table 1 (IDT DNA Technology, Coralville, IA, USA), 7.5 μL of Ultra-Pure molecular grade water (Genesee Scientific, San Diego, CA, USA), and 5 μL of DNA template. PCR was performed in a CFXOpus (Bio-Rad, Hercules, CA, USA) under the following conditions: a single hot-start cycle at 95 °C for 3 min followed by 35 cycles of denaturing at 94 °C for 15 s, annealing at 50 °C for 15 s, and extension at 72 °C for 20 s. Positive amplicons were analyzed by analysis of detectable fluorescence vs. cycle threshold values, as well as by melting curve (65 °C to 95 °C at 0.5 °C/s). Amplified products were purified and sequenced by Sanger’s method (GENEWIZ Inc., Raleigh, NC 27560, USA) and analyzed using Clustal W multi-sequence alignment (AlignX, Vector NTI Advanced 10.3.0 from Invitrogen) and compared to sequences previously deposited in the GenBank database.

## 3. Results

### 3.1. Detection of Ca. Allocryptoplasma DNA in Marine Iguanas’ Samples

Of the 85 blood DNA samples tested, 27 (31.8%) were PCR positive for one or more gene targets. Prevalence varied per location with Lobos Island having the highest prevalence (16/33, 49%), followed by San Cristobal (4/10, 40%), Española (4/14, 28.6%), and Isabela (4/18, 22%). *Ca.* Allocryptoplasma species DNA was not amplified from the 10 marine iguanas evaluated from Santa Cruz.

The 16SrRNA gene (two copies/bacterium) was by far the most successfully amplified gene, potentially due to the single copy number of *sucA*, and *rpoB* genes, as previously suggested [4].

### 3.2. Sequence Analysis

*Ca.* Allocryptoplasma sequences of 16SrRNA, *sucA*, and *rpoB* amplicons obtained from marine iguana blood DNA were deposited in the GenBank database under the following accession numbers: 16SrRNA (PZ485496-PZ485514); SucA (PZ281997-PZ282007); RpoB (PZ282008-PZ282013). A summary of sequence coverage and homologies for each gene with previously detected *Ca.* Allocryptoplasma species in hosts and vectors are referenced in Table 2. Tick species collected from marine iguanas (3 individuals) and from lava lizards (2 individuals) showed (by sequence analysis of the 12SrRNA mitochondrial DNA) 124/141 bp (88%) homology with *Amblyomma darwini* (GenBank accession ADU95851) and only 108/141 bp (76.6%) with *Amblyomma williamsi* (GenBank accession AY342271). A single base pair was found to be different in the 12SrRNA sequence among ticks collected from the lava lizards versus the marine iguanas.

**Figure 1:**
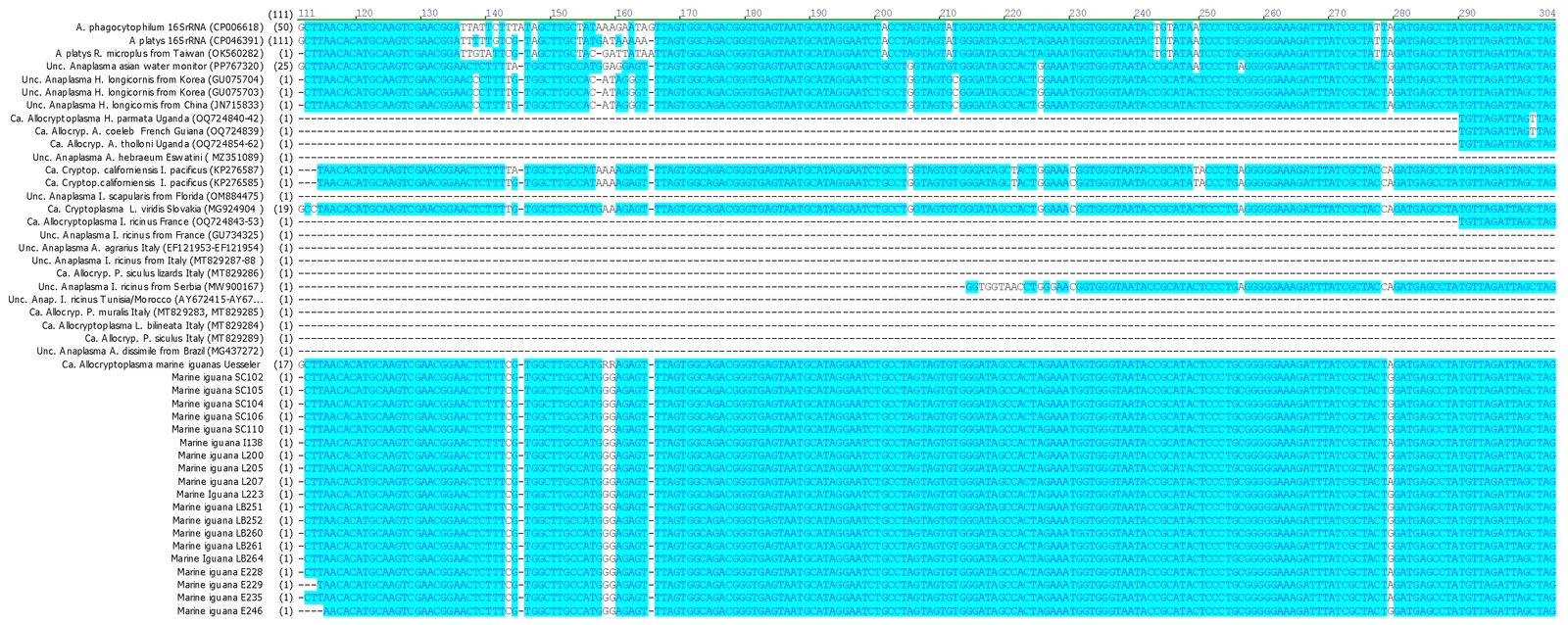

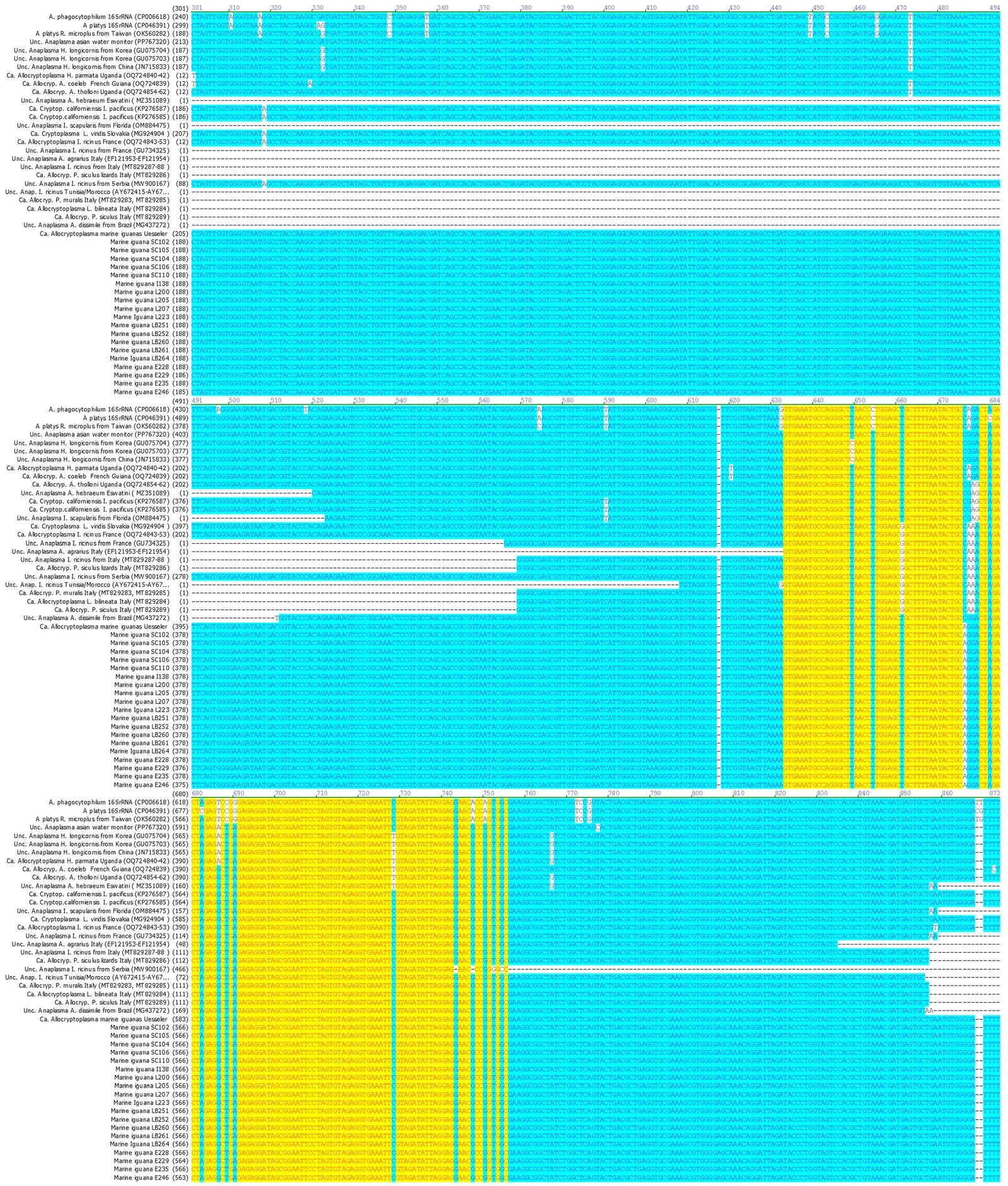

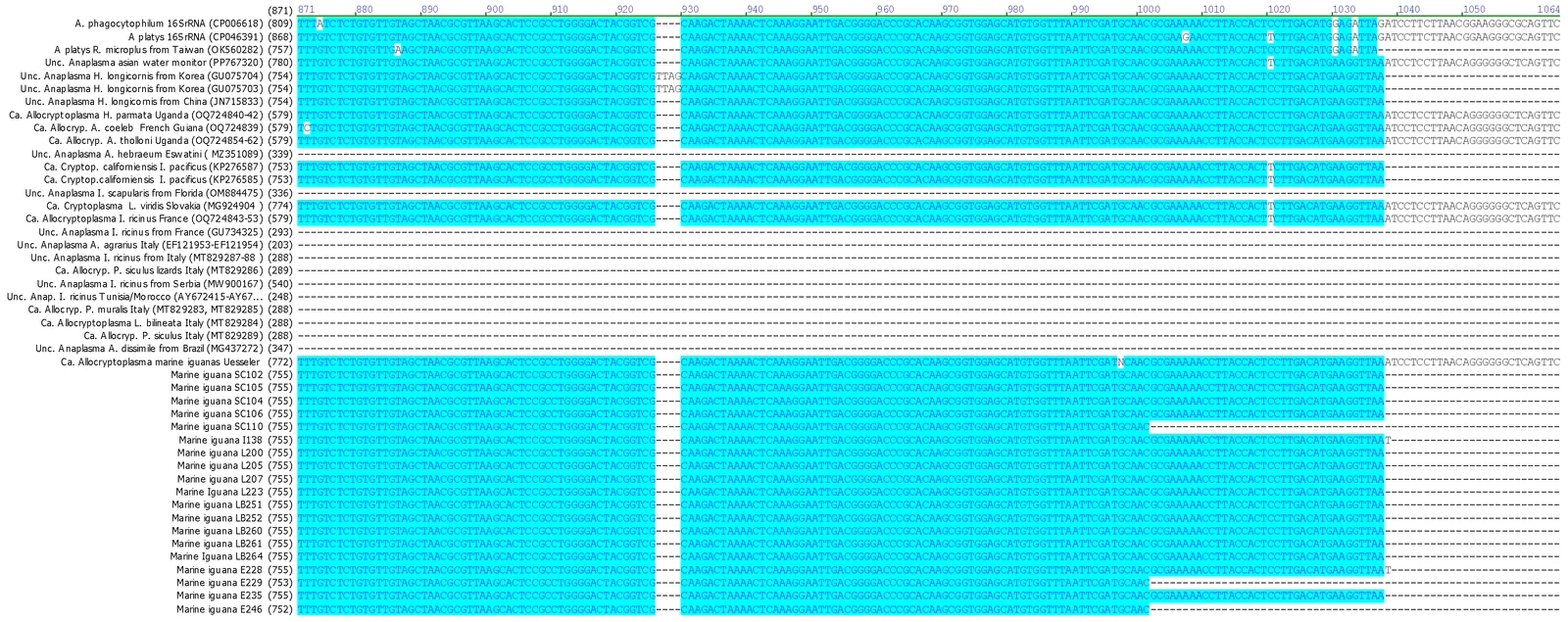
Ribosomal 16SrRNA gene sequence alignment depicting differences between sequences of previously characterized *Ca.* Allocryptoplasma (some formally described as “uncultured Anaplasma” or “*Ca.* Cryptoplasma”) and marine iguana sequences detected in this study. Note: Yellow background represents 100% homology, blue background represents high homologies. Sequences of the *Ca.* Allocryptoplasma species detected in this study have been deposited as GenBank accessions PZ485496-PZ485514.

**Figure 2:**
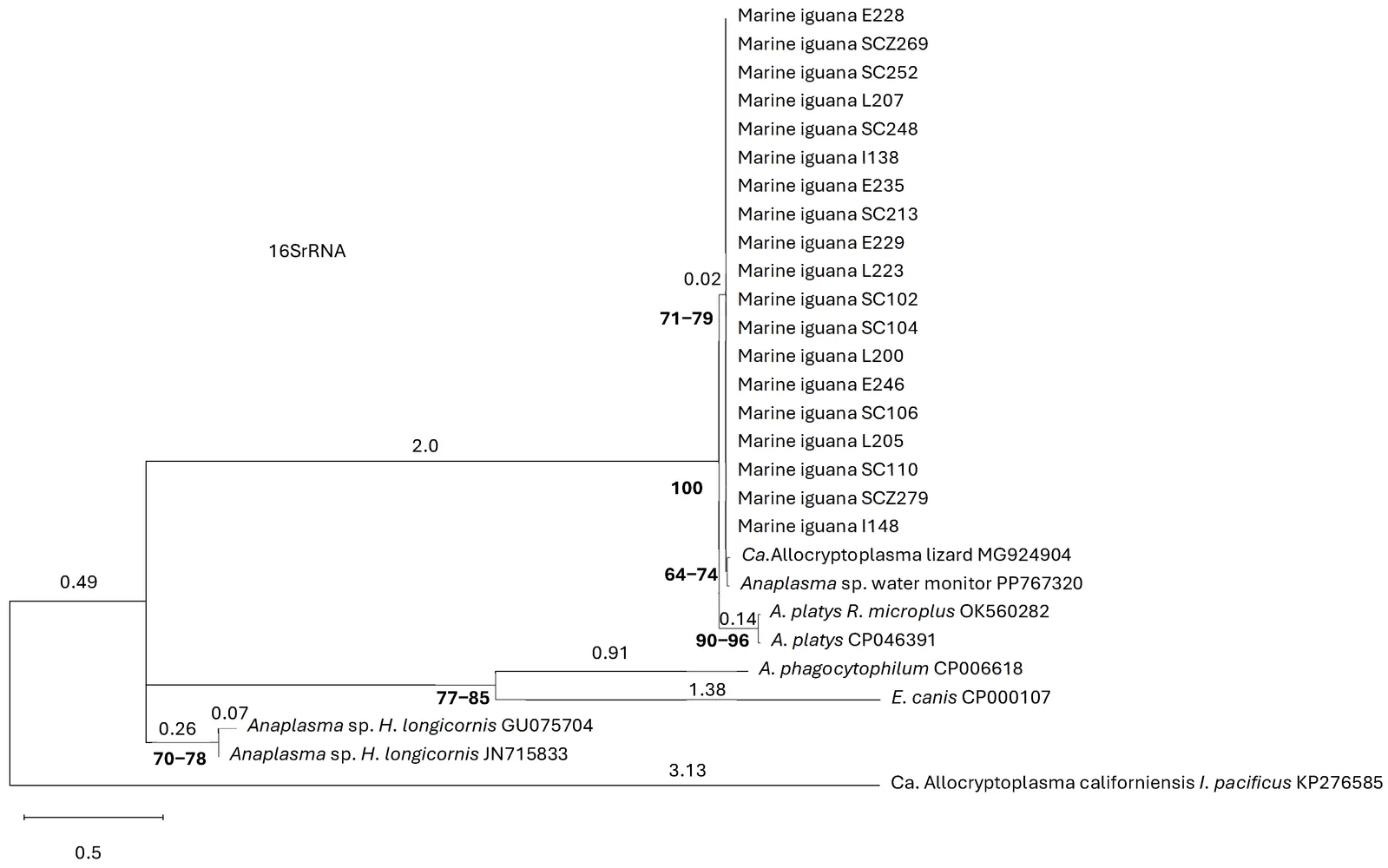
Ribosomal 16SrRNA gene sequence evolutionary analysis (the Maximum Likelihood method*) of marine iguanas *Ca.* Allocryptoplasma species. Note: Letter in sample designation denotes island origin: E = Española, I = Isabella, L = Loberia, and SC = San Cristobal. The phylogeny was inferred using the Maximum Likelihood method and the Tamura–Nei (1993) model [14] of nucleotide substitutions and the tree with the highest log likelihood (−7043.46) is shown. The tree is drawn to scale with branch lengths (branches with a distance of 0.1 or lower not shown) computed using the Maximum Likelihood method [15] and measured in the number of substitutions per site. The percentage of replicate trees in which the associated taxa clustered together, where the number of replicates (106) was determined adaptively [16], is shown next to the branches in bold. The initial tree for the heuristic search was selected by choosing the tree with the superior log-likelihood between a Neighbor-Joining (NJ) tree [17] and a Maximum Parsimony (MP) tree. The NJ tree was generated using a matrix of pairwise distances computed using the Tamura–Nei (1993) model [14]. The MP tree had the shortest length among 10 MP tree searches, each performed with a randomly generated starting tree. The analytical procedure encompassed 28 coding nucleotide sequences using 1st, 2nd, 3rd, and non-coding positions with 881 positions in the final dataset. Evolutionary analyses were conducted in MEGA12 [16] utilizing up to 4 parallel computing threads. Disclaimer: While every effort has been made to ensure the accuracy of the caption, it is provided “as is” without warranty of any kind. Users are advised to thoroughly review the caption before its use for any purpose and report any errors or issues to the authors at www.megasoftware.net. The authors and their employers disclaim any liability for damages, including but not limited to special or consequential damages. Additionally, the authors expressly disclaim all other warranties, whether expressed or implied, including the suitability of the caption text for a specific purpose, use, or application.

Sequence analysis of the *Ca.* Allocryptoplasma 16SrRNA gene (Figure 1, Table 2) revealed 881/881 bp (100%) homology with *Ca.* Allocryptoplasma previously reported in Galápagos marine iguanas [4]. There was also high sequence homology of 868/881 bp (98.5%) with *Ca.* Allocryptoplasma detected in the blood of water monitors from Thailand (GenBank PP767320) and 904/918 bp (98.5%) homology with *Ca.* Allocryptoplasma detected in lizards from Slovakia (GenBank MG924904). Sequence analysis of the *Ca.* Allocryptoplasma 16SrRNA gene from lava lizards (GenBank accession PZ492234 and PZ492235) also revealed 883/883 bp (100%) homology with *Ca.* Allocryptoplasma detected in Galápagos marine iguanas. The phylogenetic relationship to other *Ca.* Allocryptoplasma and *Anaplasma* species, based upon evolutionary analysis by the Maximum Likelihood method of the 16SrRNA gene, is shown in Figure 2.

**Figure 3:**
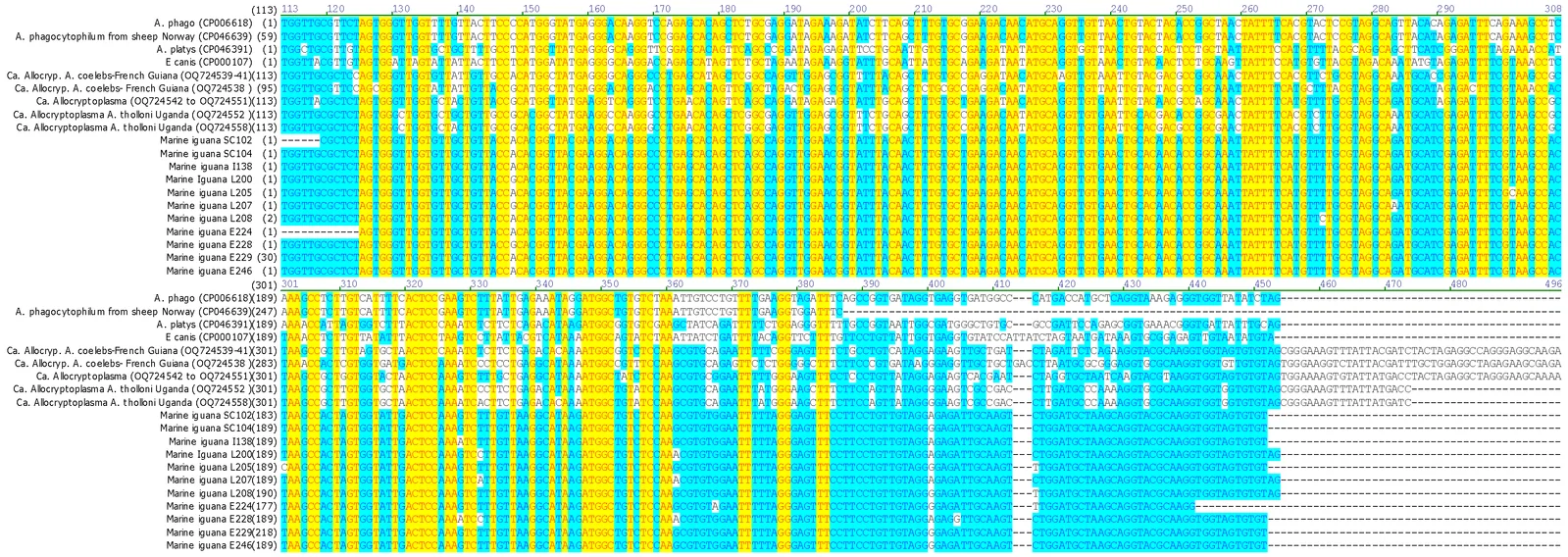
Alpha-ketoglutarate dehydrogenase (*sucA*) gene sequence depicting differences between sequences of previously characterized *Ca.* Allocryptoplasma (some formally described as “uncultured Anaplasma” or “*Ca.* Cryptoplasma”) and marine iguana’s sequences detected in this study. Note: Yellow background represents 100% homology, and blue background represents high homology. Sequences of the *Ca.* Allocryptoplasma species detected in this study were deposited as GenBank accessions PZ281997-PZ282007.

**Figure 4:**
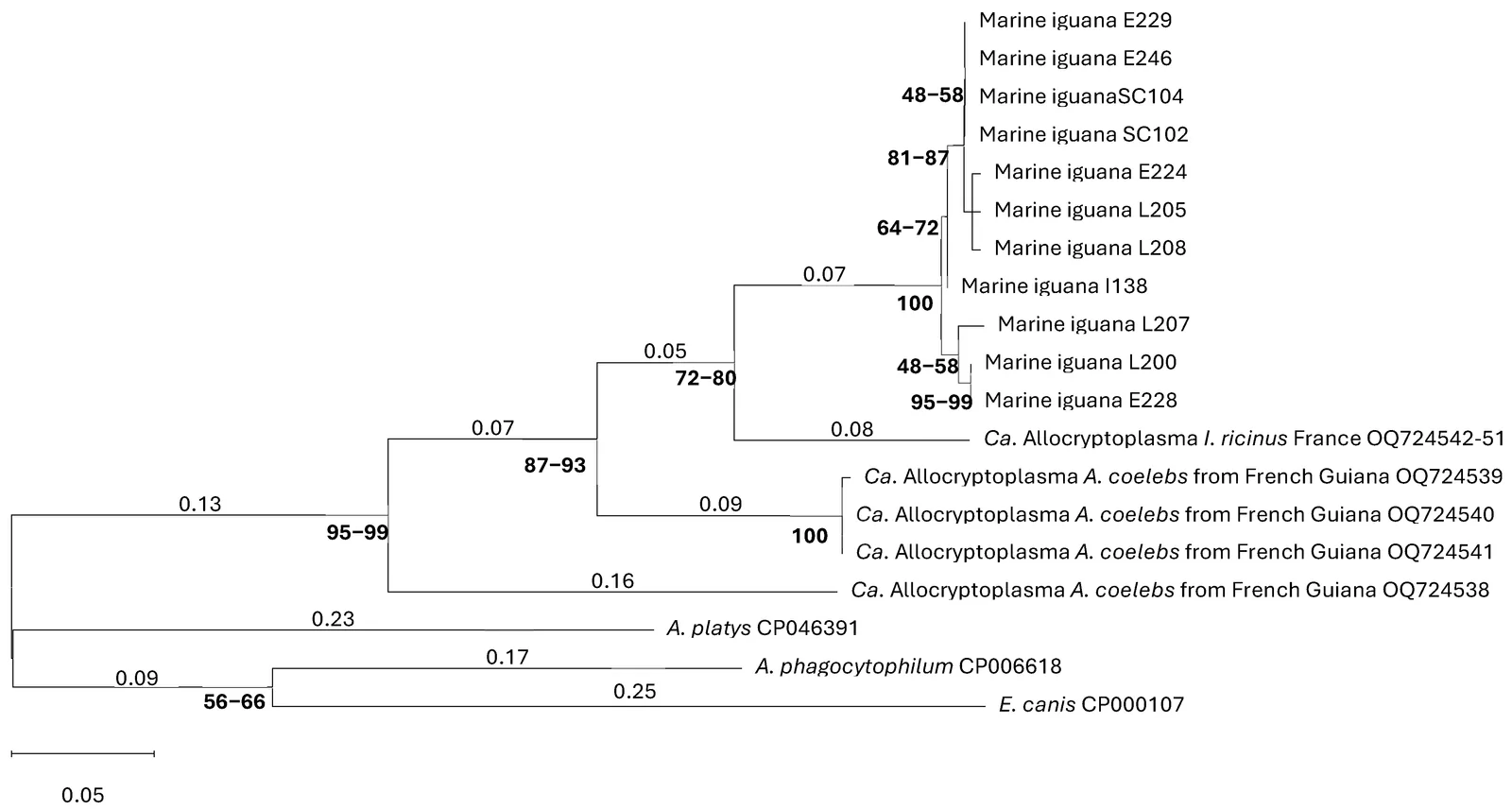
*SucA* gene sequence evolutionary analysis (the Maximum Likelihood method*) of marine iguanas *Ca.* Allocryptoplasma species. Note: Letter in sample designation denotes island origin: E = Española, I = Isabella, L = Loberia, and SC = San Cristobal. The bootstrap consensus tree inferred from 113 replicates [18] is taken to represent the evolutionary history of the taxa analyzed, where branches corresponding to partitions reproduced in less than 50% of replicate trees are collapsed. The percentage of replicate trees in which the associated taxa clustered together, where the number of replicates (113) was determined adaptively [16], is shown in bold below the branches. The initial tree for the heuristic search was selected by choosing the tree with the superior log-likelihood between a Neighbor-Joining (NJ) tree [17] and a Maximum Parsimony (MP) tree. The NJ tree was generated using a matrix of pairwise distances computed using the p-distance [15]. The MP tree had the shortest length among 10 MP tree searches, each performed with a randomly generated starting tree. The analytical procedure encompassed 19 nucleotide sequences with 336 positions in the final dataset. Evolutionary analyses were conducted in MEGA12 [16] utilizing up to four parallel computing threads.

Sequence analysis of the *sucA* gene (Figure 3, Table 2) revealed 86.6% homology (291/336 bp) with *Ca.* Allocryptoplasma in *I. ricinus* from France (GenBank accessions OQ724542-51); 82.7% homology (278/336 bp) with *Ca.* Allocryptoplasma detected in *H. parmata* from Uganda (GenBank accession OQ724541); and 77.7% homology (261/336 bp) with *Ca.* Allocryptoplasma detected in *A*. *coelebs* from French Guiana (GenBank accession OQ724538). Sequence analysis of the *Ca.* Allocryptoplasma *sucA* gene from lava lizards also revealed 336/336 bp (100%) homology with *Ca.* Allocryptoplasma detected in Galápagos marine iguanas. Evolutionary analysis by the Maximum Likelihood method of the *sucA* gene is depicted in Figure 4.

**Figure 5:**
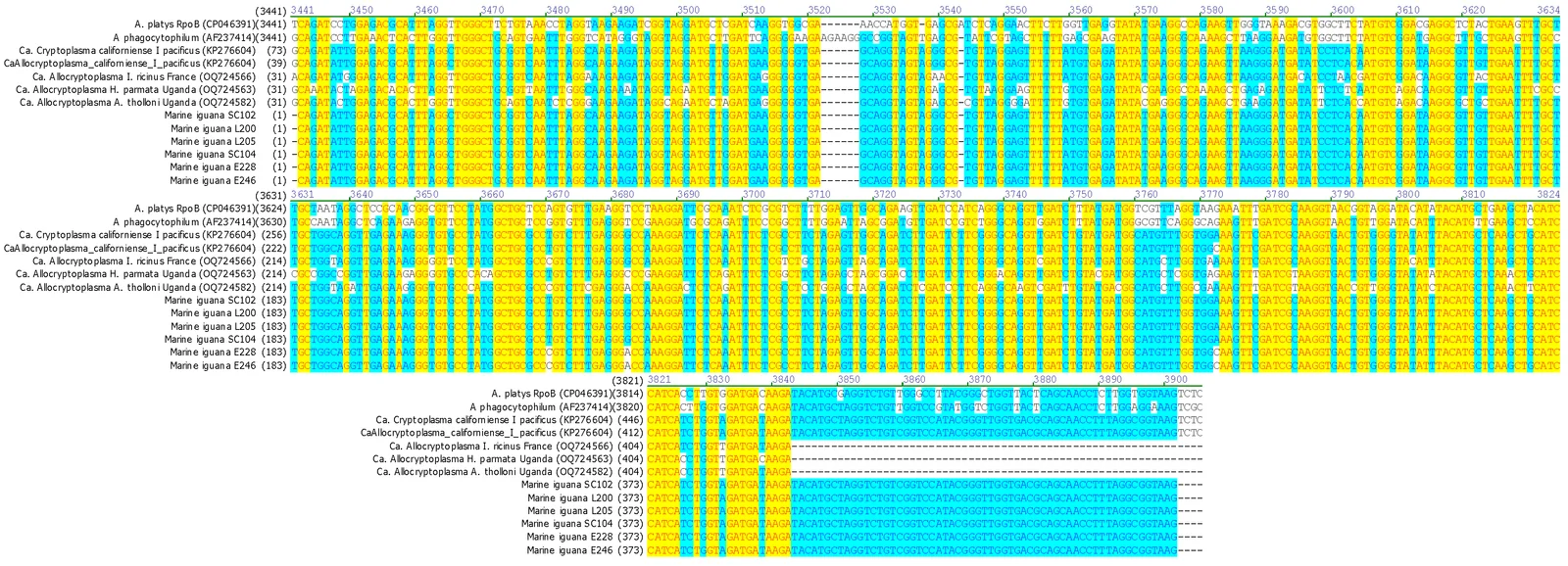
RNA polymerase subunit beta (*rpoB*) gene alignment depicting differences between sequences of previously characterized *Ca.* Allocryptoplasma (some formally described as “uncultured Anaplasma” or “*Ca.* Cryptoplasma”) and marine iguana sequences detected in this study. Note: Yellow background represents 100% homology and blue background represents high homologies. Sequences of the *Ca.* Allocryptoplasma species detected in this study were deposited as GenBank accessions PZ282008-PZ282013.

**Figure 6:**
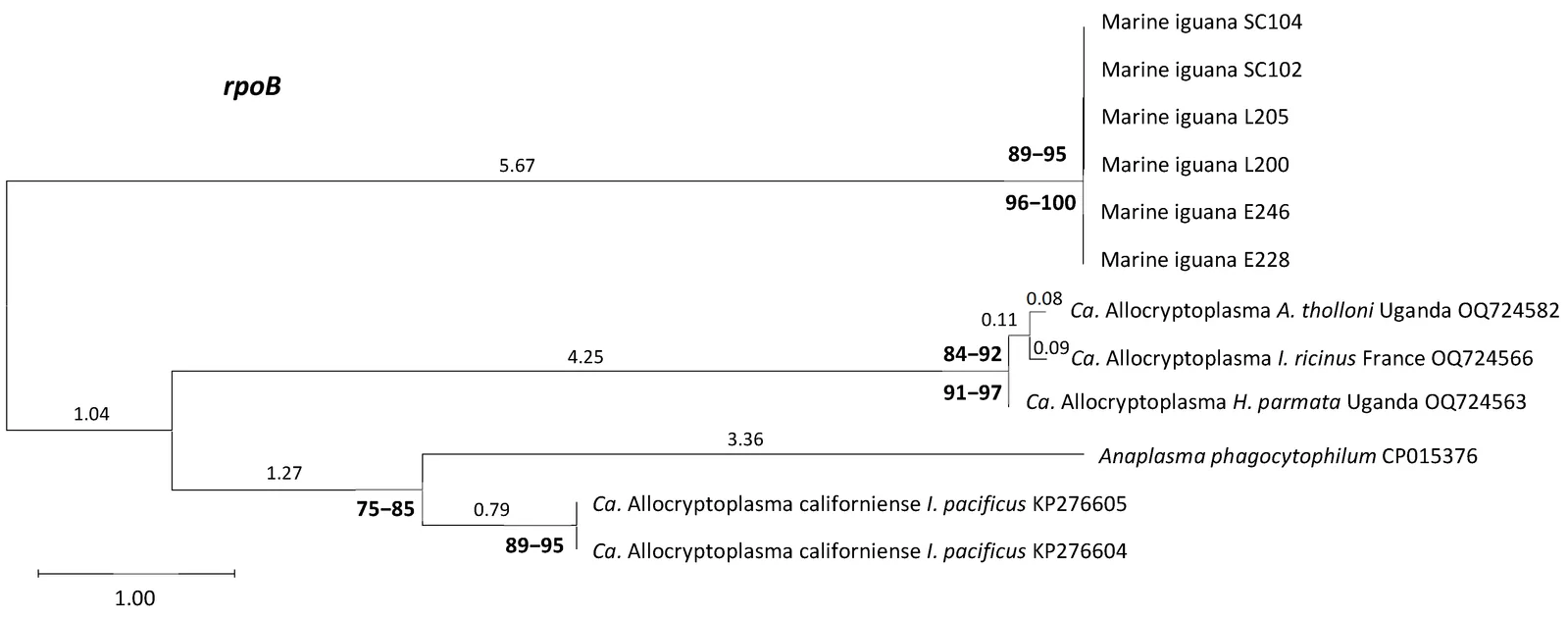
RNA polymerase subunit beta (*rpoB*) gene sequence evolutionary analysis (the Maximum Likelihood method*) of marine iguanas *Ca.* Allocryptoplasma species. Note: Letter in sample designation denotes island origin: E = Española, I = Isabella, L = Loberia, and SC = San Cristobal. The phylogeny was inferred using the Maximum Likelihood method and the Tamura–Nei (1993) model [14] of nucleotide substitutions and the tree with the highest log likelihood (−2720.71) is shown. The tree is drawn to scale with branch lengths (branches 0.01 or shorter not shown) computed using the Maximum Likelihood method [15] and measured in the number of substitutions per site. The percentage of replicate trees in which the associated taxa clustered together, where the number of replicates (78) was determined adaptively [16], is shown below in bold. The initial tree for the heuristic search was selected by choosing the tree with the superior log-likelihood between a Neighbor-Joining (NJ) tree [17] and a Maximum Parsimony (MP) tree. The NJ tree was generated using a matrix of pairwise distances computed using the p-distance [15]. The MP tree had the shortest length among 10 MP tree searches; each performed with a randomly generated starting tree. The analytical procedure encompassed 12 nucleotide sequences with 425 positions in the final dataset. Evolutionary analyses were conducted in MEGA12 [16] utilizing up to four parallel computing threads. Disclaimer: While every effort has been made to ensure the accuracy of the caption, it is provided “as is” without warranty of any kind. Users are advised to thoroughly review the caption before its use for any purpose and report any errors or issues to the authors at www.megasoftware.net. The authors and their employers disclaim any liability for damages, including but not limited to special or consequential damages. Additionally, the authors expressly disclaim all other warranties, whether expressed or implied, including the suitability of the caption text for a specific purpose, use, or application.

Sequence analysis of the *rpoB* gene (Figure 5, Table 2) revealed 100% homology (453/453 bp) and 99.6% homology (451/453 bp) with *Ca.* Allocryptoplasma detected in *I. pacificus* (GenBank accessions KP276604 and KP276605, respectively); 95.1% (431/453 bp) homology with *Ca.* Allocryptoplasma detected in *I. ricinus* from France (GenBank accession OQ724566); 87.9% (398/453 bp) homology with *Ca.* Allocryptoplasma detected in *H. parmata* from Uganda (GenBank accession OQ724563); and 87.2% (295/453 bp) homology with *Ca.* Allocryptoplasma detected in *A. tholloni* from Uganda (GenBank accession OQ724538). No *rpoB* sequences were obtained from any lava lizard tick’s DNA. Evolutionary analysis by the Maximum Likelihood method of the *RpoB* gene is shown in Figure 6.

## 4. Discussion

While whole-genome sequencing (WGS) provides the absolute gold standard for genomic classification, the lack of bacterial isolates coupled with limited sample availability and the poor sample quality (due to the long storage period between sample acquisition and processing), restricted the characterization of this bacterium to a targeted multi-gene approach. Since 16S rRNA sequence analysis alone is inadequate for resolving phylogenetic relationships at lower taxonomic level [4,19], multilocus genotyping using PCR-based sequencing analysis of 16SrRNA, *rpoB* and *sucA* housekeeping genes, which has proven successful for species-level resolution within the *Anaplasmataceae* group (including *Ca.* Allocryptoplasma) [2,4,19] was performed.

Based upon the evolutionary analyses using the Maximum Likelihood method (Figure 2, Figure 4 and Figure 6), the genetic relationships presented in this study support the presence of a novel *Ca.* Allocryptoplasma species infecting the blood of marine iguanas in the Galápagos Islands. As the first detection of this bacterium was from the blood of *Amblyrhynchus cristatus* subspecies Godzilla (a large subspecies of Galápagos marine iguana) from San Cristobal Island, the species name “*Ca.* Allocryptoplasma Godzilla sp. nov.” is proposed.

Multi-locus genetic analysis places the *Ca.* Allocryptoplasma as a newly described, genetically diverse genus of putatively tick-borne bacteria belonging to the family Anaplasmataceae, clustering alongside *Anaplasma*, *Ehrlichia*, and *Neoehrlichia* species, which are well-documented blood-borne pathogens. Its internal genetic diversity matches the thresholds seen within established pathogenic genera, confirming its status as a distinct bacterial candidate genus. Regarding its host range and enzootic circulation, *Ca.* Allocryptoplasma has been highly prevalent in lacertid lizards in Europe and in the endemic Galápagos marine iguanas (*Amblyrhynchus cristatus*). *Ca.* Allocryptoplasma has also been detected in small mammals, such as the striped field mouse (Apodemus agrarius) in Slovakia, in ticks of wild chimpanzees in Uganda, and in multiple wild bird species across the Brazilian Pantanal wetlands. As a potential vector-borne pathogen, *Ca.* Allocryptoplasma has been identified globally within diverse tick species that either infest hosts directly or share habitats. *Ca.* Allocryptoplasma has been found in *Amblyomma dissimile* and *A. coelebs* (Brazil), *A. tholloni* and *Haemaphysalis parmata* (Uganda), *I. ricinus* (France, Italy, Slovenia, Servia, Polonia, Tunisia, and Morocco), *I. scapularis* (Florida), and in *I. pacificus* in California [2,4,20].

In the Galápagos Islands, marine iguanas are commonly parasitized by four tick species, including *A. darwini*, *A. williamsii*, *Ornithodoros darwini*, and *O. galapagensis* [4,8,10,21]. Nevertheless, only *A. darwini* was identified in a reduced set of ticks collected from Galápagos marine iguanas and lava lizards in San Cristobal. Sequence analysis of *Ca.* Allocryptoplasma 16SrRNA and *sucA* genes suggest that the same *Ca.* Allocryptoplasma species that infect marine iguanas may also infect lava lizards. Interestingly, *Ca.* Allocryptoplasma sp. was not detected in the blood of Galápagos giant land tortoises (*Chelonoidis niger*) or in ticks collected from green iguanas (*Iguana iguana*) from Guayaquil, Ecuador. The detection of *Ca.* Allocryptoplasma species in *A. darwini* ticks collected from both marine iguanas and lava lizards emphasizes the need to further assess the vector capacity of this tick species.

Sequence analysis of the 16SrRNA strongly suggests that the marine iguanas *Ca.* Allocryptoplasma species differ significantly from other *Ca.* Allocryptoplasma species previously described in other reptiles: 98.5% homology with *Ca.* Allocryptoplasma detected from water monitors from Thailand (GenBank PP767320) and in lizards from Slovakia (GenBank MG924904). Sequence analysis of the 16SrRNA gene among marine iguanas from San Cristobal, Lobos, Isabella, and Española indicated 99.99% homology (916/919 bp), suggesting a limited diversity among the Galápagos islands. Reptiles (especially lizards) and their associated arthropods serve as critical, yet often underappreciated, roles in the sylvatic cycle (as reservoirs and as amplifying hosts) for several vector-borne zoonotic pathogens, including Spotted Fever Group Rickettsiae, *Borrelia* (*B. burgdorferi* and lizard-specific strains *Borrelia lusitaniae*), *Anaplasma* species, and *Trypanosoma cruzi* (Chagas disease). Even though reservoir competency varies among lizards (for example, with certain tick-borne pathogens), lizards can serve as highly competent amplifying hosts, thereby increasing the local burden of infectious agents. Lizards can also potentially serve as vectors for the transmission of pathogens to humans and other animals. Due to the relatively low (98.5%) homology with reported *Ca.* Allocryptoplasma detected in water monitors from Thailand (GenBank PP767320) and lizards from Slovakia (GenBank MG924904), the partial 16SrRNA sequences obtained from marine iguanas from the Galápagos Islands seemingly represent a novel *Ca.* Allocryptoplasma species. This conclusion is further supported by sequence analysis of two protein-coding genes, *sucA* and *rpoB* that showed 86.6% (*sucA*) and 99.6–87% (*rpoB*) similarity with Allocryptoplasma spp. sequences deposited in the GenBank database. It has been proposed that, based on the bacterial 16SrRNA gene, a 99% similarity (clustering at 1% divergence) is required for species-level resolution [22], although this value could be as low as 98.7% depending on taxa, differences between alignment methods, reference databases, and number of base pairs sequenced [23].

Amplification success of *Ca.* Allocryptoplasma from marine iguana’s blood was more successful when PCR targeted the 16SrRNA region than either the *sucA* or *rpoB* gene (with *rpoB* PCR being the least sensitive). It is not clear if this phenomenon is due to a higher copy number of the 16SrRNA-23SrRNA region or due to relatively low homologies between the primers used for this study or the limited number of reported *Ca.* Allocryptoplasma/Cryptoplasma *sucA* and *rpoB* sequences from vectors and host were deposited in GenBank.

The *sucA* gene sequences in this study showed homology of 86.6% or lower with previously reported *Ca.* Allocryptoplasma species (GenBank accessions OQ724542-51, OQ724541, and OQ724538). Homology of the *sucA* gene among marine iguanas from different islands ranged between 334/336 bp and 332/336 bp. Since in silico protein analysis of the *sucA* gene indicated 100% homology (104/104 aa) among all but one iguana from Española (99%, 103/104 aa homology), the DNA differences among marine iguanas *sucA* genes are most likely related to synonymous or silent mutations.

Similarly, sequences of the *rpoB* gene had homologies ranging from 99.6% to 87.2% with previously reported *Ca.* Allocryptoplasma species detected in tick species worldwide (GenBank accession KP276604/5; OQ724566; OQ724563; and OQ724538). Homology of the *rpoB* gene among marine iguanas from different islands ranged between 450/453 bp and 453/453 bp. In silico protein analysis of the *rpoB* gene had 100% homology (151/151 aa) among all iguana species sequenced from Española, Lobos, and San Cristobal. Unfortunately, *rpoB* sequences were not successfully sequenced from any infected marine iguana from Isabela. As in the case of *sucA*, the DNA differences among marine iguanas *rpoB* genes are most likely related to synonymous or silent mutations.

## 5. Conclusions

Using multilocus phylogenetic analysis, the data presented in this manuscript provide evidence that strongly supports the recognition of a novel *Candidatus* Allocryptoplasma lineage infecting reptiles in the Galápagos Island archipelago. Due to its close relationship with sister genera such as *Anaplasma*, *Ehrlichia*, and *Neoehrlichia*, well-documented vector-borne zoonotic pathogens, the risk of *Ca.* Allocryptoplasma as a potential zoonotic pathogen due to spill-over through small reptiles and their vectors should be strongly considered in future investigations.

## Figures and Tables

**Table 1:** Primers used for *Ca.* Allocryptoplasma 16SrRNA, *sucA*, *rpoB*, and for *Amblyomma* sp. 12SrRNA DNA amplification.

Gene Target	Primer Name	Primer Sequence
16SrRNA	Allo16S-135s	5′ CTCTTTCGTGGCTTGCCATGGGAGAGT 3′
	Allo16S-1055as	5′ GCCCCCCTGTTAAGGAGGATTTAAC 3′
*sucA*	AlloSucA-110s	5′ AGTGGTTGCGCTCTAGTGGGTTGGTG 3′
	AlloSucA-450as	5′ CTACACACTACCACCTTGGGTAC 3′
*rpoB*	AlloRpoB-74s	5′ CAGATATTGGAGACGCATTTAGGCTG 3′
	AlloRpoB-537as	5′ CCAAAATGAGACTTACCGCCTAAAG 3′
*Amblyomma* 12SrRNA	Amblyomma12SrRNA-T1	5′ AAACTAGGATTAGATACCCT 3′
	Amblyomma12SrRNA-T2	5′ AATGAGAGAGCGACGGGCGGGATGT 3′

**Table 2:** Summary of 16SrRNA, *rpoB*, and *sucA* base-pair sequence coverage and homologies (in parentheses) with previously reported *Ca.* Allocryptoplasma species in hosts and vectors.

Vectors	Location	16SrRNA	*rpoB*	*sucA*	GenBank Accessions
*Amblyomma dissimile*	Brazil	362/346 (98.8%)	-	-	MG437272
*Amblyomma coelebs*	French Guiana	733/742 (98.8%)	-	261/336 (77.7%)	OQ724538, OQ724839
*Amblyomma hebraeum*	Eswatini	332/338 (98.2%)	-	-	MZ351089
*Amblyomma tholloni*	Uganda	735/742 (99%)	295/453 (87.2%)	-	OQ724854-62, OQ724538
*Haemaphysalis longicornis*	China	900/915 (98.4%)	-	-	JN715833
*Haemaphysalis longicornis*	Korea	895/915 (97.8%)	-	-	GU075703-4,
*Haemaphysalis parmata*	Uganda	734/742 (98.9%)	398/453 (87.9%)	278/336 (82.7%)	OQ724840-42, OQ724563, OQ724541
*Ixodes pacificus*	USA (California)	896–897/915 (97.9–98%)	453–451/453 (99.6–100%)	-	KP276585, KP276587, KP276604, KP276605
*Ixodes ricinus*	Italy	283/287 (98.6%)	-	-	MT829287-88,
*Ixodes ricinus*	France	734/742 (98.9%)	431/453 (95.1%)	291/336 (86.6%)	OQ724542-51, OQ724566, OQ724843-53
*Ixodes ricinus*	Servia	518/539 (96.1%)	-	-	MW900167,
*Ixodes ricinus*	Tunisia-Morocco	242/247 (98%)	-	-	AY672415-AY672420,
*Ixodes scapularis*	USA (Florida)	330/335 (98.5%)	-	-	OM884475,
*N. autumnalis* from wall lizard (*P. siculus*)	Italy	283/288 (98.3%)	-	-	MT829286
Hosts	Location	16SrRNA	*rpoB*	*sucA*	GenBank accessions
*Apodemus agrarius* (Striped field mouse)	Italy	198/202 (98%)	-	-	EF121953-EF121954
*Podarcis muralis* (Common wall lizard)	Italy	284/288 (98.6%)	-	-	MT829283, MT829285
*Varanus salvator* (Asian water monitor)	Thailand	868/881 (98.5%)	-	-	PP767320
*L. viridis* (Green Lizard)	Slovakia	904/918 (98.5%)	-	-	MG924904
*L. bilineata* (Western green Lizard)	Italy	283/287 (98.6%)	-	-	MT829284,

## Data Availability

The data generated at the IPRL during this study are available from the corresponding author upon request.
